# Effectiveness of metabolic management centers online tools in patients with type 2 diabetes

**DOI:** 10.1080/07853890.2025.2563751

**Published:** 2025-10-09

**Authors:** Miao Xu, Jialin Li, Ying Peng, Fengmei Xu, Qidong Zheng, Yufan Wang, Tingyu Ke, Dong Zhao, Yuancheng Dai, Qijuan Dong, Bangqun Ji, Juan Shi, Yifei Zhang, Li Li, Weiqing Wang

**Affiliations:** ^a^ Department of Endocrinology and Metabolism, The First Affiliated Hospital of Ningbo University, Ningbo, China; ^b^ Department of Endocrine and Metabolic Diseases, Shanghai Institute of Endocrine and Metabolic Diseases, Ruijin Hospital, Shanghai Jiao Tong University School of Medicine, Shanghai, China; ^c^ Shanghai National Clinical Research Center for Metabolic Diseases, Key Laboratory for Endocrine and Metabolic Diseases of the National Health Commission of the P.R. China, Shanghai National Center for Translational Medicine, Ruijin Hospital, Shanghai Jiao Tong University School of Medicine, Shanghai, China; ^d^ Department of Endocrinology and Metabolism, Hebi Coal (group). LTD. General Hospital, Hebi, China; ^e^ Department of Internal medicine, The Second People’s Hospital of Yuhuan, Yuhuan, China; ^f^ Department of Endocrinology and Metabolism, Shanghai General Hospital, Shanghai Jiao Tong University School of Medicine, Shanghai, China; ^g^ Department of Endocrinology, The Second Affiliated Hospital of Kunming Medical University, Kunming, China; ^h^ Center for Endocrine Metabolism and Immune Diseases, Beijing Luhe Hospital, Capital Medical University, Beijing, China; ^i^ Department of Internal medicine of traditional Chinese medicine, Sheyang Diabetes Hospital, Yancheng, China; ^j^ Department of Endocrinology and Metabolism, People’s Hospital of Zhengzhou Affiliated Henan University of Chinese Medicine, Zhengzhou, China; ^k^ Department of Endocrinology, Xingyi People’s Hospital, Xingyi, China

**Keywords:** MMC online tools, type 2 diabetes, glycaemic control, body mass index, education level

## Abstract

**Objectives:**

To assess the value of National Metabolic Management Centers (MMC) specialized online tools, for the maintenance of metabolic control among patients with type 2 diabetes (T2DM).

**Patients:**

This retrospective study enrolled T2DM patients from 10 MMCs (June 2017–April 2021) and divided into non- and application of online tools (non-AOT and AOT) groups.

**Measurements:**

Propensity score matching (PSM) was used to balance the characteristics of patients between groups. The effect of online tools was evaluated by the change in HbA1c, with additional stratified analyses in subgroups.

**Results:**

After PSM, 12528 patients with T2DM were followed for a median of 15.88 (7.10, 24.27) months, the AOT group demonstrated better control of HbA1c (−0.90 [−2.60, 0.00] % vs. −0.70 [−2.20, 0.10] %, *p* < 0.0001), and a greater reduction in body mass index (−0.34 ± 1.68 kg/m^2^ vs. −0.13 ± 1.55 kg/m^2^, *p* < 0.0001) and Visceral fat area (VFA) (−5.33 ± 30.95 cm^2^ vs. −3.97 ± 26.11 cm^2^, *p* = 0.009), compared to the non-AOT group, and the high-frequency AOT group achieved a more significant HbA1c reduction than the low-frequency AOT group (−1.50 [−3.60, −0.30] % vs. −0.80 [−2.38, 0.10] %, *p* < 0.0001) and a greater reduction in VFA (−7.07 ± 30.32 cm^2^ vs. −4.90 ± 31.10 cm^2^, *p* = 0.010) after adjustment. Stratification analyses revealed greater HbA1c reductions in those with younger age, lower education level or poor HbA1c control at baseline.

**Conclusions:**

MMC online tools significantly improve metabolic outcomes, particularly for T2DM patients with younger age, lower education levels or poor baseline HbA1c control. They offer a scalable and effective model for out-of-hospital diabetes care.

## 1. Introduction

The prevalence and incidence of type 2 diabetes mellitus (T2DM) are increasing worldwide. According to the IDF, the global diabetes prevalence in 20–79 years old in 2021 was estimated to be 10.5% (536.6 million people), anticipated to rise to 12.2% (783.2 million) in 2045 [1]. According to recent surveys in China, the prevalence of diabetes and prediabetes in adults was 10.9%–12.8% and 35.2%–50.1%, respectively [2–4], indicating that China has the greatest burden of diabetes, making it the epicentre of the diabetes epidemic. Despite the emergence of new diabetic medications and technology, the rate of achieving ideal glycemic goals among people with T2DM remains unsatisfactory, which results in poor health outcomes. In China, among treated patients, only 39.7% had their glycated hemoglobin (HbA1c) controlled to less than 7.0% [2]. Several studies have revealed that weight loss is associated with improved insulin resistance, resulting in improved glycemic control [5,6]. A previous study revealed that among patients with prediabetes or diabetes, each additional ideal cardiovascular health metric (ICVHM), including ideal body mass index (BMI), blood pressure, HbA1c, total cholesterol (TC), etc., was associated with at least a 15% lower risk of cardiovascular disease (CVD) [7]. Therefore, we should emphasize the importance of achieving overall targets for managing patients with T2DM. Nevertheless, only 23% of patients met the composite goal of glycemic, blood pressure, and cholesterol control [8], increasing the urgency of the demand for novel therapeutic and management methods.

Online tools have been applied to increase convenience and overall access to medical care, especially to increase self-management skills in patients with chronic non-communicable diseases [9,10]. Previous studies have demonstrated the potential of mobile health tools in improving glycemic control among patients with diabetes. A meta-analysis indicates that mobile applications (apps) have a clear benefit in managing blood glucose levels in patients with T2DM, while their efficacy in type 1 diabetes (T1DM) remains uncertain. These findings underscore the importance of tailoring digital interventions to the unique needs of T2DM patients [11]. These online tools overcome time and space limitations, providing real-time feedback, self-monitoring, personalized recommendations, and out-of-hospital health education. They enhance patients’ health knowledge and awareness, promoting informed decision-making and active disease management. Rooted in behavior change theories, such as self-efficacy theory and the Health Belief Model, these tools leverage confidence-building, perceived benefits, and actionable cues to improve health behaviors.

Previous research on T2DM management has been constrained by small sample sizes [12,13], single-arm designs [14], short follow-up durations [12,15] often less than 6 months, and the absence of comprehensive stratified analyses to assess factors like education level and baseline glycemic control, which may influence engagement with digital tools. Addressing these limitations is critical for understanding how digital interventions can be optimized for T2DM management and for developing targeted strategies to improve their effectiveness across diverse patient populations.

The National Metabolic Management Centers (MMC) tools, accessible *via* the MMC app, WeChat Official Account, and web-based platform, enhance patient care through a combination of advanced technology and patient-centered features. They integrate Internet of Things (IoT) technology to enable automated uploading of blood glucose, blood pressure, and activity data from designated devices, while also allowing manual data entry, to facilitate physician-patient data sharing and teleconsultations. Additionally, the tools offer access to in-hospital test results, comprehensive health education resources, and automated follow-up reminders. Despite the growing evidence supporting the effectiveness of online tools in T2DM management, several barriers may still limit their broader applicability. Challenges such as limited digital literacy, disparities in technology access, and variability in patient engagement can affect adoption and effectiveness, particularly in older adults or populations with limited resources. MMC online tools address these issues through user-friendly interfaces, multi-platform accessibility (e.g. apps, WeChat, and web platforms), and personalized features such as automated reminders and tailored feedback, ensuring a scalable and inclusive approach to T2DM management. To address the gap in the literature, we evaluated the effect of MMC online tools application at multiple MMCs in China by comparing the metabolic parameters between the non-use and use groups at baseline and the last follow-up visit. Additionally, by analyzing stratified outcomes, we aim to determine whether these tools can reduce health disparities, particularly among patients with lower education levels or poor baseline glycemic control, and serve as a scalable model for T2DM management globally.

## 2. Methods

### 2.1. Study population and design

**Figure 1.**
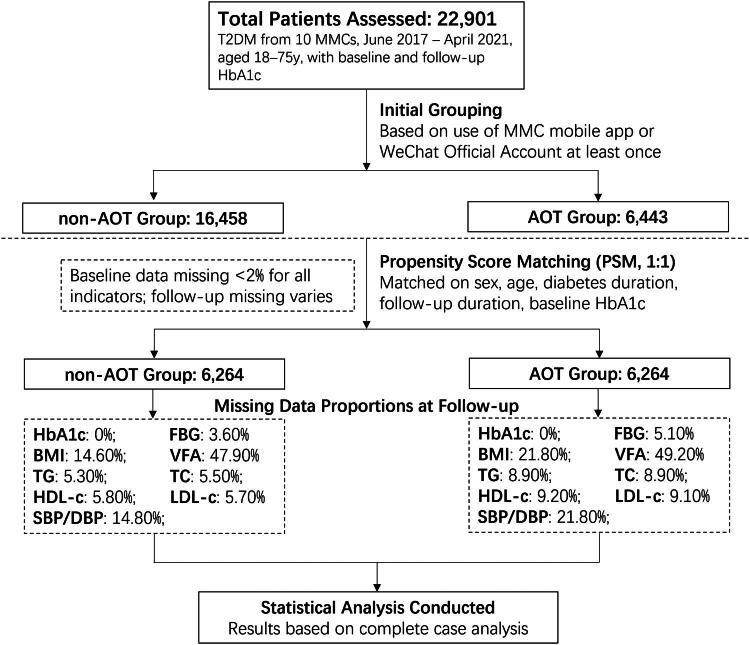
Flow diagram of the study. T2DM, type 2 diabetes; AOT, application of online tools; MMC, Metabolic Management Center; FBG, fasting blood glucose; HbA1c, glycated hemoglobin; BMI, body mass index; VFA: Visceral fat area; SBP, systolic blood pressure; DBP, diastolic blood pressure; TG, total triglyceride; TC, total cholesterol; HDL-c, high-density lipoprotein cholesterol. LDL-c, low-density lipoprotein cholesterol.

MMC is an innovative diabetes care system in China that integrates advanced medical equipment and IoT technology with traditional care patterns (ClinicalTrials.gov, number NCT03811470) [16]. As shown in Figure 1, in this retrospective observational cohort study, data were collected from 10 MMCs between June 2017 and April 2021, with all participants providing written informed consent. The study inclusion criteria were patients aged 18–75 years, visiting this MMC for the first time, and diagnosed with T2DM based on the World Health Organization (WHO) criteria (1999). We excluded patients with type 1 diabetes (T1DM), gestational diabetes (GDM), or other specific types of diabetes and those who did not return for follow-up or without HbA1c detection. In total, 22,901 adult patients with T2DM, with both baseline and the last follow-up measurement of HbA1c, were included. Standard diabetes care, as outlined in the Metabolic Disease Management Guideline for MMCs [17], was uniformly provided to all patients—both users and non-users—encompassing education, monitoring, dietary guidance, exercise plans, and medication management. Participants were categorized into an application of online tools (AOT) group if they had used the MMC mobile application or WeChat Official Account at least once during the study period (the web-based platform was not included, as it is primarily designed for healthcare professionals), while standard care procedures remained consistent across both groups. To mitigate baseline confounding, a 1:1 propensity score matching (PSM) approach was employed using baseline factors (sex, age, duration of diabetes, duration of follow-up, and baseline HbA1c level), yielding two balanced cohorts of 6264 patients each (total *n* = 12,528). Furthermore, the AOT group was divided into low-frequency and high-frequency subgroups based on the average login frequency of 23.28 times per year.

The MMC follow-up programme study protocol was approved by the ethics committee at the main center, Ruijin Hospital, Shanghai Jiao Tong University School of Medicine (2017 No.42-3), and the other participating MMCs subsequently provided approval if necessary. Informed consent was obtained from all participants. The study was conducted in accordance with the principles of the Declaration of Helsinki.

### 2.2. Data collection

The values of HbA1c, fasting blood glucose (FBG), triglyceride (TG), total cholesterol (TC), high-density lipoprotein cholesterol (HDL-c), low-density lipoprotein cholesterol (LDL-c), and other laboratory measurements were sourced from the hospital information system (HIS) and exported *via* the MMC system to minimize errors. All MMCs involved in this study are using internationally recognized methods for HbA1c and lipid profile measurements. Medical history and anthropometric data were collected by trained staff following a standardized protocol. BMI and blood pressure were measured using MMC-standardized equipment, regularly calibrated [18,19]. Visceral fat area (VFA) was measured at the level of the umbilicus using a dual bioelectrical impedance analyzer (HDS2000, Omron Healthcare Co.). To ensure real-time supervision and quality certification of MMC workflows and management standards, the MMC Experts Committee has established standardized monitoring processes and qualification systems [20]. All data were gathered *via* the MMC-specialized system, with baseline data recorded at the first visit and follow-up data at the most recent visit before April 2021, reflecting the latest available records. Home monitoring data, whether uploaded automatically through MMC-compatible devices or entered manually, were used only for feedback purposes, but excluded from the analysis.

### 2.3. MMC Online tools

**Figure 2.**
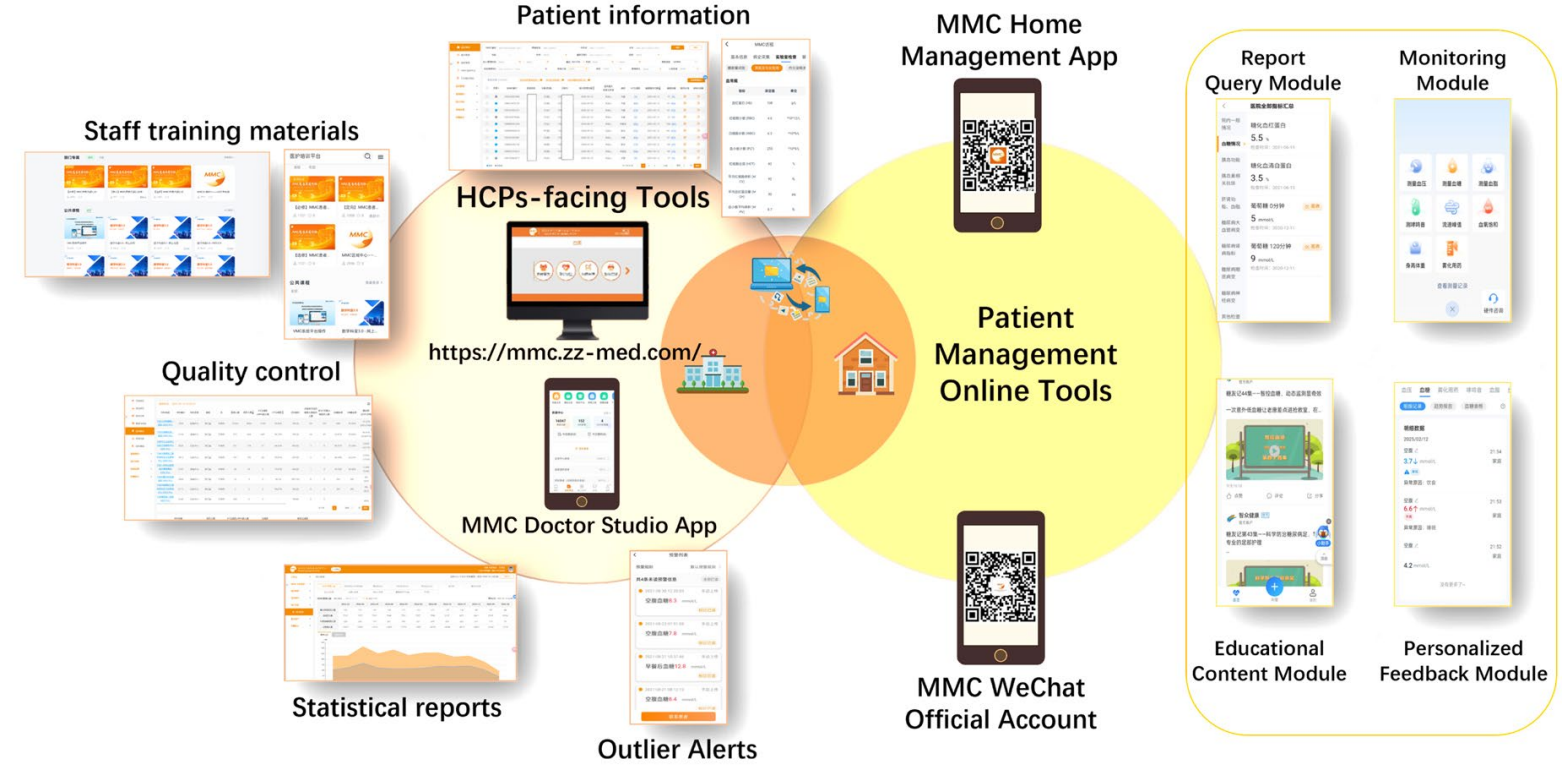
Contents and features of the MMC online tools. MMC, Metabolic Management Center.

Each patient with a smartphone was assisted by MMC staff to install the MMC Home Management App, compatible with both iOS and Android systems. Patients were guided to enable notifications from the WeChat Official Account, received detailed training, ongoing support during hospital visits, and access to instructional videos on hospital televisions to boost engagement. The MMC online tools are designed to provide comprehensive support for T2DM management, such as health literacy enhancement, home-based health monitoring, self-management empowerment, etc. While the online tools designed for healthcare professionals (HCPs), offered functionalities such as patient information management, staff training, quality control, and statistical analysis (Figure 2, see Supplementary Materials for details).

### 2.4. Statistical analysis

Statistical analyses were performed using SPSS version 22.0 (Chicago, IL, USA). Data are presented as median and interquartile range (IQR) for continuous variables and as frequency (%) for categorical variables. Missing data were handled using a complete case analysis approach, without imputation, and data missingness for outcomes is detailed in Figure 1. Data were tested for normal distribution and logarithmically transformed for statistical analysis, when required. Nonparametric test was used for continuous variables. General linear regression model was used to compare the between-group reduction of HbA1c adjusting for major covariables including sex, age, education level, duration of diabetes, region, family income, FBG, HbA1c, TG, TC, BMI and SBP. We also used restricted cubic spline regressions to model associations between online tools frequency and changes in HbA1c levels. All p values were 2-tailed, and a p value < 0.05 was considered statistically significant.

## 3. Results

### 3.1. Baseline clinical characteristics of the participants

In total, 22,901 patients with T2DM were enrolled in our study, of whom 13202 (57.6%) were men. The mean age of these patients was 54.3 ± 11.3 years, and the median diabetes duration was 5.33 (0.92, 11.00) years. Male patients were more likely to use online tools than female patients. Patients in non-AOT group were much older and had a longer duration of diabetes, were less likely to have completed high school, and exhibited higher baseline blood glucose levels (all *p* < 0.0001) (Supplementary Table 1). After PSM, 12528 patients were included in the analysis, with 6264 enrolled in the AOT group. There were no significant differences in sex, age, duration of follow-up, or baseline HbA1c between the two groups; however, the duration of diabetes was still slightly longer in non-AOT group than that in AOT group (4.25 [0.50, 10.08] vs. 4.00 [0.25, 10.17], *p* = 0.035). Compared to patients with an education level below high school, those with an education level at high school and above were more likely to use online tools. Patients in AOT group had slightly lower FBG levels than those in non-AOT group at baseline (8.19 [6.74, 10.41] vs. 8.39 [6.77, 11.00], *p* = 0.001) (Table 1).

**Table 1.** Profiles of enrolled patients and the characteristic of T2DM patients in different groups after PSM.

	Total	non-AOT group	AOT group	*p* value
No. of participants	12528	6264	6264	
Age, year	50.59 ± 11.63	50.64 ± 11.51	50.54 ± 11.76	0.611
Male sex, *n* (%)	8023 (64.00%)	4009 (64.00%)	4014 (64.08%)	0.926
Online tools login frequency (times/year)	0.12 (0, 2.16)	/	2.16 (0.72, 15.48)	/
Duration of diabetes, years	4.17 (0.33, 10.08)	4.25 (0.50, 10.08)	4.00 (0.25, 10.17)	0.035
Duration of follow-up (months)	15.88 (7.10, 24.27)	16.07 (7.08, 24.33)	16.03 (7.20, 24.30)	0.313
Education level-High school and above, n (%)	5759 (54.00%)	2719 (43.40%)	4050 (64.70%)	<0.0001
Region				<0.0001
East China	54.50%	55.10%	54.00%	
Central China	13.10%	6.10%	20.10%	
North China	16.80%	16.40%	17.30%	
Southwest China	15.50%	22.40%	8.60%	
Family income (CNY/year)				<0.0001
<100,000	49.10%	56.00%	42.30%	
100,000–300,000	26.80%	25.30%	28.20%	
>300000	7.80%	5.30%	10.20%	
unknown	16.30%	13.30%	19.40%	
BMI (kg/m^2^)	26.06 ± 3.84	26.08 ± 3.84	26.03 ± 3.85	0.522
VFA (cm^2^)	102.12 ± 42.09	101.44 ± 41.68	102.81 ± 42.49	0.208
SBP (mmHg)	130.81 ± 17.78	131.39 ± 18.32	130.17 ± 17.15	<0.0001
DBP (mmHg)	78.18 ± 11.37	78.32 ± 11.46	78.03 ± 11.28	0.196
FBG (mmol/L)	8.29 (6.76, 10.69)	8.39 (6.77, 11.00)	8.19 (6.74, 10.41)	0.001
HbA1c (%)	8.10 (6.80, 9.90)	8.10 (6.80, 9.80)	8.07 (6.80, 9.90)	0.975
TG (mmol/L)	1.66 (1.14,2.57)	1.69 (1.15,2.62)	1.64 (1.13,2.50)	0.009
TC (mmol/L)	4.93 ± 1.33	4.96 ± 1.36	4.90 ± 1.30	0.023
HDL-c (mmol/L)	1.17 ± 0.32	1.18 ± 0.33	1.16 ± 0.31	0.009
LDL-c (mmol/L)	2.97 ± 0.98	2.97 ± 1.01	2.97 ± 0.96	0.920

Note: Variables with normal distribution were presented as mean and standard deviation (SD), variables with skewed distribution were presented as median and interquartile range (IQR), and continuous variables were analyzed by independent-sample t-test or nonparametric test, respectively. Proportions were compared using the χ^2^ test. *p* < 0.05 was considered statistically significant.

Abbreviations: T2DM, type 2 diabetes; PSM, propensity score matching; AOT, application of online tool; BMI, body mass index; VFA, Visceral fat area; SBP, systolic blood pressure; DBP, diastolic blood pressure; FBG, fasting blood glucose; HbA1c, glycated hemoglobin. TG, triglyceride; TC, total cholesterol; HDL-c, high-density lipoprotein cholesterol; LDL-c: low-density lipoprotein cholesterol.

### 3.2. Changes from baseline in HbA1c between AOT and non-AOT groups, low frequency AOT group and high frequency AOT group

**Figure 3.**
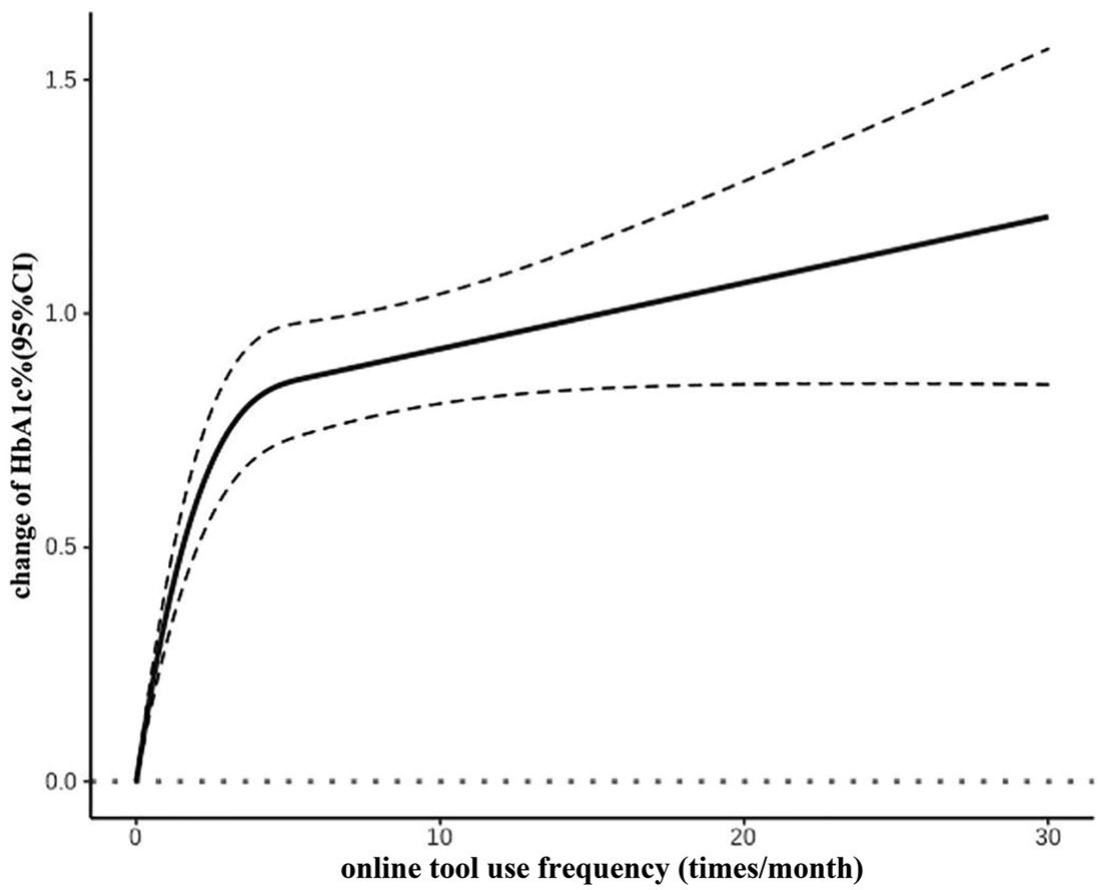
Restricted cubic spline regressions modeling associations between change of HbA1c and online tools use frequency. HbA1c, glycated hemoglobin; CI, confidence interval

According to the backend database after PSM, compared to non-AOT group, the patients in AOT-group achieved a greater decrease in HbA1c (−0.90 [−2.60, 0.00] % vs. −0.70 [−2.20, 0.10] %, *p* < 0.0001) after adjusting for major covariables (Table 2). Among the patients in AOT group, the mean frequencies of online tools logging in low-frequency AOT sub-group or high-frequency AOT sub-group were 3.6 or 99.6 times/year, respectively. Those with high-frequency AOT use achieved a greater decrease in HbA1c levels (−1.50 [−3.60, −0.30] % vs. −0.80 [−2.38, 0.10] %, *p* < 0.0001) after adjusting for major covariables (Table 3). Furthermore, we used restricted cubic spline regressions to model the associations between changes in HbA1c levels and online tools use frequency. Regression splines showed a nonlinear positive relationship between changes in HbA1c levels and the frequency of online tools use (Figure 3).

**Table 2.** The comparison of patients with T2DM between with or without online tools before and after MMCs management.

	Non-AOT group			AOT group			
	Baseline	Follow-up	Change from baseline	Baseline	Follow-up	Change from baseline	p* value
FBG (mmol/L)	8.39 (6.77, 11.00)	7.30 (6.37, 8.94)	−0.69 (−2.85, 0.76)	8.19 (6.74, 10.41)	7.21 (6.25, 8.63)	−0.70 (−2.80, 0.71)	0.470
HbA1c (%)	8.10 (6.80, 9.80)	6.90 (6.30, 7.90)	−0.70 (−2.20, 0.10)	8.07 (6.80, 9.90)	6.70 (6.10, 7.60)	−0.90 (−2.60, 0.00)	<0.0001
BMI (kg/m^2^)	26.08 ± 3.84	25.95 ± 3.73	−0.13 ± 1.55	26.03 ± 3.85	25.70 ± 3.64	−0.34 ± 1.68	<0.0001
VFA (cm^2^)	101.44 ± 41.68	97.47 ± 40.15	−3.97 ± 26.11	102.81 ± 42.49	97.48 ± 38.68	−5.33 ± 30.95	0.009
SBP (mmHg)	131.39 ± 18.32	129.94 ± 16.99	−1.45 ± 18.09	130.17 ± 17.15	129.55 ± 16.33	−0.61 ± 17.49	0.453
DBP (mmHg)	78.32 ± 11.46	77.02 ± 10.38	−1.30 ± 10.86	78.03 ± 11.28	77.20 ± 10.63	−0.83 ± 10.83	0.019
TG (mmol/L)	1.69 (1.15, 2.62)	1.54 (1.07, 2.29)	−0.13 (−0.68, 0.26)	1.64 (1.13, 2.50)	1.46 (1.01, 2.19)	−0.13 (−0.64, 0.26)	0.093
TC (mmol/L)	4.96 ± 1.36	4.65 ± 1.19	−0.31 ± 1.34	4.90 ± 1.30	4.62 ± 1.18	−0.28 ± 1.30	0.009
HDL-c (mmol/L)	1.18 ± 0.33	1.26 ± 0.40	0.08 ± 0.38	1.16 ± 0.31	1.22 ± 0.33	0.06 ± 0.27	0.297
LDL-c (mmol/L)	2.97 ± 1.01	2.64 ± 0.93	−0.33 ± 1.02	2.97 ± 0.96	2.67 ± 0.91	−0.30 ± 0.96	0.443
visit rate (visits/y)		2.50 ± 1.36			2.89 ± 1.34		<0.0001

Note: Variables with normal distribution were presented as mean ± standard deviation (SD), variables with skewed distribution were presented as median and interquartile range (IQR)Data was shown as mean ± standard deviation or median (interquartile range). p*: comparison of metabolic change between AOE and non-AOE groups performed using a general linear regression model, adjusting for sex, age, duration of diabetes, duration of follow-up, education level, region, family income, and SBP, HbA1c, TG, and TC at baseline. *p* < 0.05 was considered statistically significant.

Abbreviations: T2DM, type 2 diabetes; MMC, Metabolic Management Center; AOT, application of online tools; FBG, fasting blood glucose; HbA1c, glycated hemoglobin; BMI, body mass index; VFA: Visceral fat area; SBP, systolic blood pressure; DBP, diastolic blood pressure; TG, triglyceride; TC, total cholesterol; HDL-c, high-density lipoprotein cholesterol; LDL-c, low-density lipoprotein cholesterol.

**Table 3.** The comparison of patients with T2DM between with low or high frequency AOT usage before and after MMCs management.

	Low frequency AOT group			High frequency AOT group			
	Baseline	Follow-up	Change from baseline	Baseline	Follow-up	Change from baseline	p* value
FBG (mmol/L)	8.18 (6.73, 10.26)	7.30 (6.30, 8.81)	−0.61 (−2.54, 0.82)	8.26 (6.76, 10.80)	6.98 (6.10, 8.00)	−1.07 (−3.48, 0.33)	<0.0001
HbA1c (%)	8.00 (6.80, 9.80)	6.80 (6.20, 7.70)	−0.80 (−2.38, 0.10)	8.30 (6.80, 10.20)	6.40 (5.90, 7.00)	−1.50 (−3.60, −0.30)	<0.0001
BMI (kg/m^2^)	26.06 ± 3.82	25.72 ± 3.60	−0.34 ± 1.61	25.95 ± 3.94	25.63 ± 3.79	−0.32 ± 1.92	0.530
VFA (cm^2^)	103.33 ± 42.83	98.43 ± 38.67	−4.90 ± 31.10	100.73 ± 41.08	93.66 ± 38.52	−7.07 ± 30.32	0.010
SBP (mmHg)	130.49 ± 17.15	129.72 ± 16.34	−0.77 ± 17.46	128.91 ± 17.09	128.92 ± 16.32	−0.01 ± 17.60	0.607
DBP (mmHg)	77.84 ± 11.22	77.21 ± 10.51	−0.63 ± 10.71	78.80 ± 11.50	77.16 ± 11.12	−1.64 ± 11.27	0.015
TG (mmol/L)	1.65 (1.14, 2.53)	1.49 (1.03, 2.21)	−0.11 (−0.63, 0.27)	1.62 (1.13, 2.38)	1.34 (0.94, 2.07)	−0.13 (−0.64, 0.26)	<0.0001
TC (mmol/L)	4.88 ± 1.29	4.62 ± 1.20	−0.26 ± 1.29	5.00 ± 1.32	4.62 ± 1.10	−0.38 ± 1.36	0.016
HDL-c (mmol/L)	1.17 ± 0.31	1.22 ± 0.33	0.05 ± 0.27	1.15 ± 0.30	1.23 ± 0.32	0.08 ± 0.27	<0.0001
LDL-c (mmol/L)	2.94 ± 0.95	2.66 ± 0.92	−0.28 ± 0.94	3.09 ± 0.98	2.71 ± 0.88	−0.37 ± 1.00	0.069
visit rate (visits/y)		2.77 ± 1.27			3.36 ± 1.51		<0.0001

Note: Data was shown as mean ± standard deviation or median (interquartile range). p*: comparison of metabolic change between low-frequency AOE group and high-frequency AOE group performed using general linear regression model, adjusting for sex, age, duration of diabetes, duration of follow-up, education level, region, family income, and SBP, HbA1c, TG, and TC at baseline. *p* < 0.05 was considered statistically significant.

Abbreviations: T2DM, type 2 diabetes; AOT, application of online tools; MMC, Metabolic Management Center; FBG, fasting blood glucose; HbA1c, glycated hemoglobin; BMI, body mass index; VFA: Visceral fat area; SBP, systolic blood pressure; DBP, diastolic blood pressure; TG, total triglyceride; TC, total cholesterol; HDL-c, high-density lipoprotein cholesterol. LDL-c, low-density lipoprotein cholesterol.

### 3.3. Changes from baseline in other metabolic indexes between AOT and non-AOT groups, low frequency AOT group and high frequency AOT group

Table 2 showed the decrease in BMI and VFA were greater in AOT group than non-AOT group (BMI: −0.34 ± 1.68 kg/m^2^ vs. −0.13 ± 1.55 kg/m^2^, *p* < 0.0001; VFA: −5.33 ± 30.95 cm^2^ vs. −3.97 ± 26.11 cm^2^, *p* = 0.009). However, no significant difference was observed between the high-frequency AOT group and the low-frequency AOT group for BMI changes, while the difference in VFA reduction remained significant (−7.07 ± 30.32 cm^2^ vs. −4.90 ± 31.10 cm^2^, *p* = 0.010) (Table 3). For other metabolic indexes, although statistically significant differences were observed in the changes of DBP and TC between the AOT and non-AOT groups, and in DBP, TG, TC, and HDL-c between the high-frequency and low-frequency AOT groups, the magnitude of these differences was small and of limited clinical significance. The AOT group had a statistically higher visit rate than the non-AOT group (2.89 ± 1.34 vs. 2.50 ± 1.36 visits/year, *p* < 0.0001) (Table 2), with a similar trend observed between high- and low-frequency users (3.36 ± 1.51 vs. 2.77 ± 1.27 visits/year, *p* < 0.0001) (Table 3).

### 3.4. Effect of online tools on HbA1c change in subgroups

**Figure 4.**
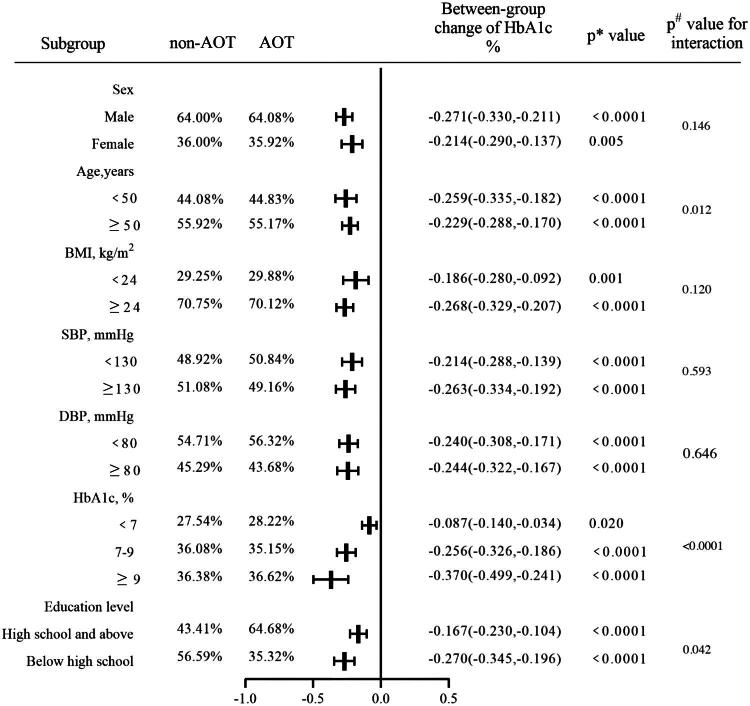
Subgroup analyses of the association between with or without online tools and between-group change of HbA1c. Data was presented as the mean (standard deviation) or median (interquartile range). *: Comparison of metabolic changes between AOT and non-AOT groups performed using a general linear regression model, adjusting for sex, age, duration of diabetes, duration of follow-up, education level, SBP, HbA1c, TG, and TC at baseline. ^#^: Interaction test performed using general linear regression model, adjusting for sex, age, duration of diabetes, duration of follow-up, education level, region, family income, SBP, HbA1c, TG, and TC at baseline. p < 0.05 was considered statistically significant. AOT, application of online tools; FBG, fasting blood glucose; HbA1c, glycated hemoglobin; BMI, body mass index; VFA: Visceral fat area; SBP, systolic blood pressure; DBP, diastolic blood pressure; HDL-c, high-density lipoprotein cholesterol. LDL-c: low-density lipoprotein cholesterol.

To investigate the effect of online tools on HbA1c levels, we further conducted a stratified analysis (Figure 4). The results showed that the decrease in HbA1c in AOT group was 0.087%, 0.256%, and 0.370% greater than that in non-AOT group after adjusting for confounders in subgroups with baseline HbA1c < 7%, 7%–9%, and ≥ 9%, respectively (*p* for interaction < 0.0001). Stratified analysis by age revealed that the AOT group significantly reduced blood glucose levels in both the <50 years (−0.259 [−0.335, −0.182] %) and ≥50 years subgroups (−0.229 [−0.288, −0.170]%), the effect was more pronounced in the <50 years subgroup compared to the ≥50 years subgroup (*p* for interaction = 0.012). Analyses stratified by education level showed that a significant between-group reduction in HbA1c was observed in both subgroups, with a greater reduction in patients with education level below high school (−0.270 [−0.345, −0.196] %) compared to those with high school or above (−0.167 [−0.230, −0.104] %) (*p* for interaction = 0.042). There were no significant interactions among the subgroups of sex, BMI, and blood pressure after adjusting for major covariates (*p* for interaction > 0.05, Figure 4).

## 4. Discussion

In this multicenter, real-world metabolic study, we compared changes in metabolic indexes under the management of 10 MMCs in China and found that metabolic parameters were significantly improved. Those who used online tools showed a greater decrease in HbA1c levels and BMI than those who did not. Patients who took full advantage of online tools achieved the best HbA1c improvement, indicating that the use of online tools may have offered an auxiliary method for the care of people with diabetes.

Although the prevalence of diabetes continues to increase, only 25.8% of patients received treatment, and only 39.7% of those treated achieved ideal glycemic control [2]. This unsatisfactory control status leads to a higher incidence of diabetes-associated chronic complications, thus increasing the economic burden [21,22]. This could be due to inadequate awareness of diabetes, serious imbalance in the ratio of physicians to patients, or disparities in treatment skills. To address these challenges, MMC was founded in 2016. By integrating advanced medical equipment with IoT technology, MMCs are committed to creating an online and offline combined management model for metabolic disorders, including diabetes and obesity, to achieve a more convenient and precise model of patient care. In this study, compared to baseline data, patients achieved all-around improvements in glucose, lipid profiles, and body weight control, demonstrating that MMC is a promising alternative for metabolic disease management. Study from Zhao et al. [23] revealed that decreases in HbA1c, TG, and HDL-c levels were significantly greater in the MMC group than in the control group, which was similar to our results. They also found that patients under MMC management possessed a better understanding of diabetes.

The evolution of health-related applications has played a powerful role in disease prevention and management from large-scale public health issues to personal medical care [24,25]. The high usability and acceptability of smartphones makes media platforms a promising solution. Diabetes is a chronic clinical condition most commonly targeted by the mobile app industry. A progressively increasing number of medical applications have recently emerged to meet the needs of patients with diabetes [26]. Previous studies have shown that with smartphone apps, patients with diabetes achieved better glycemic control without increasing hypoglycemic event rates [27,28]. MMCs are multiple network resources, such as MMC home management apps, WeChat, and web-based platform, helping extend medical care outside hospitals. These resources are highly accessible and make it easy to share information in real time on smartphones. By using these resources, patients could easily access popular scientific knowledge and thematic lectures given by endocrinologists. Patients could upload their daily data, such as blood glucose and blood pressure, and steps into the app so that they could adjust their monitoring and lifestyle intervention programmes according to their own indicators. Patients were encouraged to self-educate by searching MMC app where articles were renewed regularly, also leading to better glucose control. To assess the function of these online tools, we classified the patients into two groups according to their usage of online tools. We observed obviously different characteristics among patients who did and did not use the tools. Pre-PSM, the non-AOT group included older patients with longer diabetes duration, lower education levels, and a different gender distribution compared to the AOT group. Although PSM balanced some characteristics, residual demographic influences, particularly age, may affect engagement and outcomes. Stratified analyses (Figure 4) indicate a significant age-related interaction, but the intervention’s effectiveness remains significant across all subgroups, highlighting a potential area for further exploration in future studies to address this variation.

The decreases in HbA1c, BMI, and VFA levels were much greater in patients who used online tools than in those who did not, even after adjusting for possible confounding factors. In the AOT group, a reduction of 0.9% in HbA1c was observed, surpassing the average 0.49% improvement reported in a meta-analysis of 14 similar studies from 1996 to 2015 [11], trials in India [14] and Spain [29] demonstrated smaller improvements in HbA1c. Several small-sample studies have suggested a greater reduction in HbA1c [30,31], these studies included only around 100 participants and were based on strict RCT design [30] or even single-arm study [31], and involved participants with higher baseline HbA1c levels. Such designs may impose highly controlled patient management, since patient engagement is the primary factor affecting the impact of these tools, excessive external intervention may artificially enhance engagement, potentially overestimating the actual effectiveness of the tools in real-world settings. Although, modest reductions in BMI, particularly the decrease in VFA, can significantly improve insulin sensitivity and overall metabolic health [32,33]. These improvements hold promise for lowering the risk of long-term diabetes complications and healthcare costs. However, other parameters, such as lipid profiles and blood pressure, exhibited limited or no significant differences between the non-AOT and AOT groups. Within the AOT group, comparison between low-frequency and high-frequency users suggested a dose-dependent effect of the online tools on these indicators, though the improvements remained modest. These findings indicate that the MMC online tools may have a more pronounced impact on glycemic control, while their effects on lipid profiles and blood pressure are more variable, potentially indicating a need for tailored interventions to address broader metabolic outcomes. Additionally, the presence of missing follow-up data for some indicators, particularly VFA, where the completion rate was notably low (as detailed in Figure 1), may reduce statistical power due to the decreased sample size, and these results should be interpreted with caution.

Notably, compared to the low-frequency AOT group, the high-frequency AOT group showed further improvements in glycemic indicators, with an average HbA1c reduction of 1.5% and an FBG decrease of 1.07 mmol/L, both of which were significantly greater and could potentially corresponds to a substantial reduction in the risk of both microvascular and macrovascular complications [34]. A higher frequency of online tool usage was associated with greater improvements in HbA1c, suggesting that the observed glycemic improvements were driven by the utilization of these digital tools. Traditional care often relies on in-person visits and scheduled appointments, which can limit patient engagement and timely intervention. MMC tools enhance accessibility, particularly for patients in remote or underserved areas, such as real-time feedback, health education push notifications, and automated reminders, which help to better patient self-monitoring, increased adherence to treatment protocols, and enhanced diabetes education. While most digital tools used in previous studies also incorporated monitoring and educational feedback functions [11], the MMC online tools integrate multiple modules and connect in-hospital and out-of-hospital data. Additionally, they provide regular medication and follow-up reminders, bridging the gap between patient self-management and hospital-based care, which may explain their glycemic control effects.

We also found that patients with younger age, lower education levels and poorer baseline HbA1c control experienced more pronounced improvements when using the MMC online tools. There are several potential explanations. First, individuals with less formal education may have previously lacked access to consistent diabetes education and self-management resources, thus benefiting substantially from the structured, user-friendly digital interfaces offered by MMC. As in diabetic patients with low education levels in Mexico City, HbA1c levels decreased from 9.3% to 6.7% after receiving diabetes self-management education (DSME), indicating that structured diabetes education can significantly improve glycemic control [35]. Second, those with higher baseline HbA1c likely had greater scope for improvement, so even a moderate enhancement in their adherence or lifestyle behaviors could yield larger observable gains in glycemic control, this result is consistent with several studies [31,36]. Third, the ease of receiving automated reminders and step-by-step health guidance may have been particularly valuable for patients who had limited prior exposure to chronic disease management strategies. Nonetheless, our study did not explicitly measure digital literacy, self-efficacy, or psychological distress, which may mediate the relationship between education level, baseline glycemic status, and intervention effectiveness. Future research incorporating qualitative interviews or psychosocial assessments could help refine these interventions to optimize engagement and maximize benefits for vulnerable patients.

A crucial aspect of this study is discerning whether the observed benefits were due to the online tools themselves or to a potential confounding effect of greater overall care adherence among users. To address this, we analyzed the frequency of in-person visits and found that the AOT group had a statistically higher visit rate than the non-AOT group, with a similar trend observed between high- and low-frequency users. While this difference is statistically significant, we contend that it is not primarily a source of confounding bias but rather a direct outcome of the digital intervention’s effectiveness. First, the absolute difference in visit frequency is clinically modest, amounting to less than one additional visit every two years per patient, which is unlikely to single-handedly drive the substantial metabolic improvements observed. More importantly, the MMC online tools are engineered to enhance patient engagement through features like automated reminders for follow-up appointments and continuous health data monitoring. These functionalities actively encourage adherence to the entire standardized care pathway prescribed by the MMC. Therefore, the slightly higher visit frequency is likely a consequence of the intervention prompting better engagement, rather than a reflection of a pre-existing difference in patient motivation. This interpretation is consistent with the goal of digital health tools, which is to synergize with and enhance traditional care. This finding suggests that the MMC online tools successfully bridge the gap between hospital visits, reinforcing the importance of the comprehensive, standardized care model and leading to better outcomes.

Our multi-center design enhanced the representativeness of the study within the Chinese healthcare context. We included 10 MMCs distributed across diverse regions—including eastern coastal areas (Ningbo, Shanghai, Yuhuan, Yancheng), central cities (Zhengzhou, Hebi), southwestern cities (Kunming, Xingyi), and the northern capital (Beijing)—thereby spanning both major urban centers and smaller, less urbanized locales. This geographical coverage allowed us to capture variations in healthcare infrastructure, resource availability, and patient demographics, which may differ substantially between coastal megacities and inland regions. As a result, although our findings primarily reflect the national experience in China, they may be more broadly generalizable than data derived from a single-site study. Nonetheless, we acknowledge that certain remote or minority-populated areas remain underrepresented; thus, future prospective research could further investigate how differences in cultural acceptance, technological adoption, and policy support might influence the real-world effectiveness of digital health interventions in other regions or countries.

The MMC online platform has been in use since 2016, primarily promoted by MMC centers to patients with diabetes and other metabolic disorders. MMC users can also recommend these tools to family and friends, which has contributed to a broad user base. Currently, there are more than 2,000 MMC sub-centers throughout mainland China, representing the country’s largest diabetes management model and spanning urban to rural regions. The user interface was designed to be simple and user-friendly, and we plan to introduce specialized layouts for older adults or individuals with low digital literacy in future iterations. Since the completion of the present study, new features such as online expert consultations and personalized lifestyle guidance have been introduced, and incorporated artificial intelligence (AI) technologies into diabetes management to meet the diverse needs of patients across varying socioeconomic backgrounds and health literacy levels.

This study had several limitations. First, due to technical constraints at the time of data collection, we only recorded whether patients logged into the MMC apps or WeChat and their overall login frequency, without distinguishing which specific features (e.g. educational modules, real-time monitoring, and feedback reminders) they accessed most frequently. Consequently, we could not determine which components were most influential in improving glycemic control. However, following the completion of this study, we have upgraded our system to monitor module-level usage, enabling us to capture how patients interact with various functionalities in greater detail. Future investigations can leverage these enhanced capabilities to examine metrics such as time spent on individual modules or types of engagement, thereby identifying and refining the features that yield the greatest clinical benefits. By comparing differential interaction across apps, WeChat, and other digital tools, researchers can further tailor eHealth solutions to meet the diverse needs of different patient populations. Second, although our large, multi-center dataset helps minimize certain biases, due to the retrospective observational design, we were unable to systematically track patients who did not return for follow-up or record specific reasons for their dropout. This also meant that we did not capture potential negative outcomes, such as poor adherence related to technological barriers, and could not assess how these factors may have contributed to patient attrition. Future prospective research incorporating discontinuation rates investigation, structured adverse event reporting and detailed documentation of dropout reasons will be essential for fully evaluating both the benefits and potential challenges of digital health interventions in T2DM management. Third, this study focused on quantitative outcomes from electronic medical records and did not include qualitative assessments; the absence of psychological and behavioral measures limits our insight into factors driving the effectiveness of the MMC tools. Future studies should adopt qualitative methodologies, including patient surveys or interviews, to complement quantitative data and enhance understanding of the usability and acceptability of digital health interventions. Online tools should be widely used by patients at MMCs and should be taught to make most of the functions of the apps. Therefore, we should be cautious in interpreting the current findings, as this study cannot establish causal conclusions due to its observational design lacking randomization, and further research is needed to fully explore the value of online tools in diabetic management.

In conclusion, this study highlights the effectiveness of online tools in managing T2DM, improving glycemic control and weight. These tools provide a valuable alternative for out-of-hospital care, especially for patients with younger age, low education or poor HbA1c control. To integrate them into routine care, scaling across healthcare systems is essential, with the potential to reduce long-term complications and healthcare costs. Future research should focus on the long-term impact, feature optimization, and strategies to increase adoption, particularly among older or less tech-savvy patients.

## Supplementary Material

Supplementary material.docx

Supplementary Table 1.docx

## Data Availability

The data that support the findings of this study are available upon request from the corresponding author, but they are not publicly available because of privacy restrictions.
