# Supplementary material for: Effectiveness of metabolic management centers online tools in patients with type 2 diabetes
Source: Ann Med. 2025 Oct 9;57(1):2563751. doi: 10.1080/07853890.2025.2563751 (PMC12517409; doi:10.1080/07853890.2025.2563751)
Supplement: Supplementary material.docx [file IANN_A_2563751_SM5918.docx]

**Detailed Exposition of the Metabolic Management Center Online Tools**

1. Overview of the Metabolic Management Center (MMC) Online Tools

The MMC online tools were developed to support out-of-hospital care for patients with type 2 diabetes mellitus (T2DM). The platforms consist of patient-facing tools, accessible via the MMC mobile app or WeChat Official Account through QR code scanning. Healthcare professionals (HCPs) -facing tools, including a web-based platform accessible at <https://mmc.zz-med.com/> and a dedicated mobile app for HCPs, called MMC Doctor Studio App. These integrated platforms aim to enhance patient self-management through educational resources and personalized recommendations, and supporting HCPs with patient information management, staff training materials, quality control, and statistical reports, collectively improving diabetes management outcomes. (figure 2)


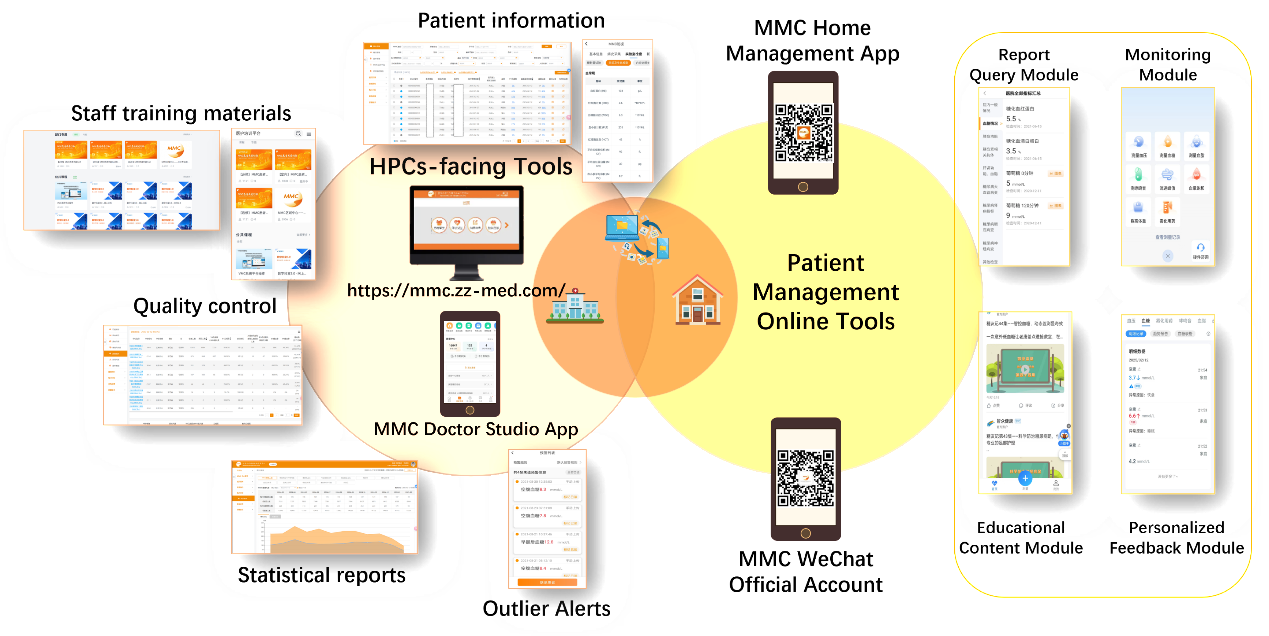


1. Key Functionalities
   1. Patient-Facing Tools
2. Report Query Module: This module allows patients to access and review their laboratory results, such as HbA1c, lipid profiles (e.g., triglycerides, total cholesterol, HDL-c, LDL-c), and other metabolic markers, directly within the app. Results are presented in a clear, user-friendly format, enabling patients to understand their progress in glycemic and metabolic control, and fostering motivation and awareness of their health status.
3. Educational Content Module: Health literacy enhancement through educational materials (articles, short videos) authored by endocrinology specialists, with regular content updates ensuring clinical accuracy.
4. Monitoring Module: Home-based health monitoring enabling real-time uploads of glucose levels, blood pressure, and lifestyle data via IoT-enabled devices or manual input, to facilitate physician-patient data sharing and teleconsultations.
5. Automated Reminders: Self-management empowerment via automated reminders for follow-up notifications, medication adherence, dietary control, and physical activity schedules.
   1. HCPs-facing Tools, accessible via the web platform at <https://mmc.zz-med.com/> and MMC Doctor Studio App, provide the following functionalities:
6. Patient Information Management: This module provides centralized access to patients’ profiles, enabling HCPs to query questionnaire data (entered by both patients and healthcare professionals, such as lifestyle habits or symptom reports) and laboratory test results (e.g., HbA1c, lipid profiles) extracted from hospital databases, available on both web and app interfaces.
7. Staff Training Materials: the online curriculum incorporates lectures by experts in the field of endocrine and metabolic disorders, covering SOP training, guideline interpretation, expert experience sharing, and academic article writing. Through these structured educational modules, healthcare providers can achieve an indepth understanding of diabetes management principles and MMC's operational workflows.
8. Quality Control: The MMC network conducts a comprehensive assessment and ranking of every center through a multidimensional scoring system.
9. Statistical Reports: The web platform’s statistical functionality dynamically displays key metrics, including the number of managed patients, the prevalence of diabetic complications (e.g., diabetic retinopathy, nephropathy), the achievement rates of clinical targets (e.g., HbA1c < 7%, blood pressure < 130/80 mmHg), and the trends of these metrics over time, enabling HCPs to understand the overall management status of patients at the center and improve care strategies.
10. Outlier Alerts: The app platform provides automated alerts for abnormal patient data, including measurements taken both in-hospital and out-of-hospital, notifying HCPs of patients with abnormal metrics and enabling direct communication through the app for guidance (when patients are also using the MMC app).

For further details on the MMC online tools, readers are referred to the article titled *Medical Education in Medical Education in Diabetes Management on the New Horizon: Insights From Metabolic Management Center* in *Journal of Diabetes* (2025; 17(3):e70075, doi:10.1111/1753-0407.70075) and the study’s registration on ClinicalTrials.gov (NCT03811470).
