# Supplementary material for: Effectiveness of metabolic management centers online tools in patients with type 2 diabetes
Source: Ann Med. 2025 Oct 9;57(1):2563751. doi: 10.1080/07853890.2025.2563751 (PMC12517409; doi:10.1080/07853890.2025.2563751)
Supplement: Supplementary Table 1.docx [file IANN_A_2563751_SM5917.docx]

**Supplementary Table 1. Profiles of enrolled patients and the characteristic of patients in groups according to MMC-specialized online tools use frequency**

|  | Total | non-AOT group | AOT group | p value |
| --- | --- | --- | --- | --- |
| No. of participants | 22901 | 16458 | 6443 |  |
| Age, year | 54.3±11.3 | 56.0±10.5 | 50.1±12.0 | <0.0001 |
| Male sex, n (%) | 13202 (57.6) | 9060 (55.0) | 4142 (64.2) | <0.0001 |
| Duration of diabetes, years | 5.33 (0.92, 11.00) | 5.92 (1.33, 11.67) | 3.83 (0.17, 10.00) | <0.0001 |
| Duration of follow-up (months) | 16.70 (7.57, 24.83) | 17.03 (7.80, 25.17) | 15.55 (7.07, 24.16) | <0.0001 |
| Education level-High school and above, n (%) | 10145 (44.3) | 5952 (36.2) | 4193 (65.1) | <0.0001 |
| BMI (kg/m2) | 26.0±3.8 | 25.9±3.7 | 26.1±4.0 | <0.0001 |
| VFA (cm2) | 101.4±41.2 | 101.1±41.2 | 102.1±41.3 | 0.508 |
| SBP (mmHg) | 132.1±18.6 | 132.9±19.0 | 130.1±17.4 | 0.109 |
| DBP (mmHg) | 77.5±11.5 | 77.2±11.5 | 78.1±11.3 | 0.525 |
| FBG (mmol/L) | 8.43 (6.81, 10.97) | 8.50 (6.85, 11.20) | 8.17 (6.73, 10.39) | <0.0001 |
| HbA1c (%) | 8.20 (6.90, 10.00) | 8.30 (7.00, 10.00) | 8.00 (6.68, 9.90) | <0.0001 |
| Triglyceride (mmol/L) | 1.61 (1.11, 2.45) | 1.60(1.10,2.43) | 1.64(1.13,2.50) | 0.011 |
| Total cholesterol (mmol/L) | 4.92±1.31 | 4.93±1.32 | 4.90±1.29 | 0.606 |
| HDL cholesterol (mmol/L) | 1.20±0.34 | 1.21±0.35 | 1.16±0.31 | 0.001 |
| LDL cholesterol (mmol/L) | 2.96±1.00 | 2.96±1.01 | 2.97±0.95 | 0.794 |

22,901 diabetes patients from 10 MMCs in China. Data are given as mean with standard deviation (SD) or median with interquartile range (IQR) or n (%). Comparisons of mean values and proportions were performed using independent-sample t test or nonparametric test or the χ2 test, respectively.

Abbreviations: MMC, Metabolic Management Center; AOT, application of online tools; BMI: body mass index; VFA: Visceral fat area; SBP: systolic blood pressure; DBP: diastolic blood pressure; FBG: fasting blood pressure; HbA1c: glycated haemoglobin. HDL cholesterol: high density lipoprotein cholesterol. LDL cholesterol: low density lipoprotein cholesterol.
